# Influence of Sociodemographic Factors on Patient Completion of Patient-Reported Outcome Measures in Hand Surgery Patients

**DOI:** 10.1016/j.jhsg.2025.100872

**Published:** 2025-10-30

**Authors:** Ezra Goodrich, Tobin Smith, Mithil Gudi, Nebiyat M. Girma, Carl Wilson, Charles S. Day

**Affiliations:** ∗ Department of Orthopedic Surgery, Henry Ford Health System, Detroit, MI; † Wayne State University School of Medicine, Detroit, MI; ‡ Michigan State University College of Human Medicine, Grand Rapids, MI

**Keywords:** Area deprivation index, Hand, Patient-reported outcomes, Sociodemographic factors, Wrist

## Abstract

**Purpose:**

Patient-reported outcome measures (PROMs) are standardized surveys that assess health outcomes, symptoms, functional status, and quality of life. Although prior studies have evaluated completion and/or participation across orthopedic subspecialties, no current studies have examined both completion and participation outcomes specifically in hand surgery. This study investigates the association between sociodemographic factors and PROM completion and participation in hand surgery patients.

**Methods:**

A retrospective review was conducted of patients who underwent carpal tunnel release, trigger finger release, or both by an orthopedic hand surgeon at an academic center between 2020 and 2024. Demographic variables, area deprivation index, and PROM participation and completion were recorded. Participation was defined as completing at least one pre- and one postoperative survey, and completion was defined as completing all pre- and postoperative surveys. Univariable and multivariable logistic regression were used to identify factors associated with PROM completion and participation.

**Results:**

In total, 406 patients were included for review. Black patients had lower rates of survey completion, and both Asian and Black patients demonstrated reduced participation of PROM surveys. Furthermore, patients from minority backgrounds and patients residing in areas with greater sociodemographic disadvantage were less likely to complete and participate in PROM surveys. Additionally, increasing age was also associated with decreased likelihood of both survey completion and participation.

**Conclusions:**

This study found PROM completion and participation rates to be lower among hand surgery patients of Black race, racial/ethnic minorities, higher area deprivation index score, and older age. However, only participation rates were lower for Asian race and those with active smoking status. These findings support similar studies in other orthopedic subspecialties and support the need for equitable PROMs collection to better represent patients across all sociodemographic backgrounds.

**Type of study/level of evidence:**

Prognostic III.

Patient-reported outcome measures (PROMs) are standardized questionnaires completed by patients, which gather information concerning health outcomes, symptoms, health-related quality of life, and functional status.^1^ Although PROMs were originally developed for research purposes, they have since been adopted into the clinical setting to assist with clinical decision making, evaluate cost-effectiveness, drive quality improvement, and establish insurance indications for treatment.^1^^,^^2^ For PROMs to represent and benefit patients universally, their clinical utility should be considered against the burden patient’s experience when completing them.^3^ Studies have found that several barriers exist that prevent patients of lower sociodemographic statuses from completing PROMs.^4^ Within patients that complete PROMs, studies show that sociodemographic markers of deprivation were associated with lower long-term completion rates.^5^, ^6^, ^7^ One such marker of sociodemographic deprivation is area deprivation index, a validated area-based index of socioeconomic disadvantage based on Census indicators of education, employment, housing quality, and poverty.^8^ Additional markers of sociodemographic status include age, sex, race, insurance status, marital status, primary language spoken, and smoking status, to name a few.

In a retrospective review of 219,891 hand surgery patients across six orthopedic subspecialties including foot and ankle, hand, spine, trauma, arthroplasty, and oncology, Bernstein et al^4^ found that patients without online electronic medical record (EMR) portal access, those of Black race, with Medicare insurance, nonmarried, or non-English speaking status were less likely to complete PROMs surveys and were underrepresented in aggregate PROMs data. However, this study did not perform multivariable analyses for individual subspecialties and therefore did not evaluate sociodemographic associations with completion/participation rates within each subspecialty. Other studies evaluated PROMs completion within total joint arthroplasty and orthopedic spine surgery, respectively, and found similar sociodemographic associations.^2^^,^^6^ Prior studies of PROMs scores within hand surgery patients undergoing trigger finger or carpal tunnel release evaluated correlations between socioeconomic status and PROMs scores.^9^^,^^10^ These studies found lower income level, lower education level, higher sick leave, immigrant status, being widowed, and dependence on social assistance to be associated with worse PROMs scores. However, these studies did not evaluate the relationship between sociodemographic status and outcome scores participation and completion rates.

The purpose of our study was to determine the association between PROMs survey completion and participation rates and sociodemographic factors for hand surgery patients. Our primary hypothesis was that there would be an association between sociodemographic factors and PROMs survey completion and participation rates. Our secondary hypothesis was that associations in hand surgery would mirror those reported for other orthopedic subspecialties.

## Materials and Methods

We conducted a retrospective review of patients who underwent carpal tunnel release, trigger finger release, or a combination of both procedures by a fellowship trained orthopedic hand surgeon at an academic health system between January 2020 and December 2024. Carpal tunnel release and trigger finger release were selected given their use in pre-existing studies of PROMs in hand surgery, and these procedures have an expected number of before surgery and postoperative visits and PROMs collection timepoints. Patients were included if they underwent carpal tunnel release, trigger finger release, or both during this collection window and were English speaking. Patients were excluded if they did not undergo one of these procedures or were non-English speaking, as the health system only offered English language PROMs surveys at the time of the study. Patient demographic variables (as recorded at the time or directly around the time of surgery) were recorded for each patient, including sex, age, body mass index (BMI), area deprivation index (ADI), race, ethnic background, minority status, marital status, insurance status, and smoking status. Patients were considered to be of minority status if they were non-White race or Hispanic ethnicity.

The ADI state deciles were determined based on the patient’s home address, using the University of Wisconsin Neighborhood Atlas.^11^ A state decile of 1 indicates least disadvantaged and a state decile of 10 indicates most disadvantaged. State deciles were recorded for each patient and aggregated into three groups for ease of statistical analysis. Group 1 included state deciles 1–3, group 2 included state deciles 4–6, and group 3 included state deciles 7–10. The PROM surveys used for this study were the National Institutes of Health Patient-Reported Outcomes Measurement Information System Upper Extremity, Patient-Reported Outcomes Measurement Information System Pain Interference, and Quick Disabilities of the Arm, Shoulder, and Hand. Patients were asked to fill out surveys prior to all before surgery and postoperative visits through the online portal. If questionnaires were not filled out prior to a visit, patients also had the option to fill them out on an iPad at their visit. Patients were considered to participate in PROMs if they filled out at least one questionnaire before and one questionnaire after surgery and were considered to complete PROMs if they filled out all pre- and postoperative questionnaires. These criteria were chosen based on prior studies reporting similar criteria.^6^ Univariable and multivariable logistic regressions were performed to identify factors associated with PROMs completion and participation. SAS Version 9.4 (Cary,) and R software were used to perform the summarization, analysis, visualization, and tabular presentation of the data. This research was approved by the Institutional Review Board (approval no. 16587-01). As this was a retrospective chart review, informed consent was not required.

## Results

A total of 612 patients were identified in the initial study population. Eleven patients were excluded because of non-English language and 195 patients were excluded because of patient encounters outside of the window of PROMs collection. In total, 406 patients were included for review. Demographic data are summarized in the Table 1. Continuous variables are summarized as means with SD. Of the 406 patients, 192 (47%) completed all PROMs, and 363 (89%) participated in PROMs. Seventeen patients (4.2%) were lost to follow-up and had incomplete PROMs, but two of these patients participated in PROMs by completing at least one before surgery PROMs survey. The remaining 389 patients all attended at least one postoperative appointment.

**Table 1:** Demographics

Demographic Variable	N = 406
Sex, n (%)	
Female	249 (61.3)
Male	157 (38.7)
Age, mean (SD)	62.1 (13.25)
BMI, mean (SD)	31.9 (8.11)
ADI^3^, n (%)	
Group 1	162 (40.3)
Group 2	83 (20.6)
Group 3	157 (39.1)
Decline	4
Race, n (%)	
White	231 (60.6)
Black	136 (35.7)
Asian	11 (2.9)
Other	3 (0.8)
Decline	25
Ethnic Background, n (%)	
Non-Hispanic/Latino	366 (97.1)
Hispanic/Latino	11 (2.9)
Decline	29
Minority Status, n (%)	
Nonminority	235 (59.8)
Minority	158 (40.2)
Decline	13
Marital Status, n (%)	
Single	103 (25.8)
Married	226 (56.6)
Divorced	44 (11.0)
Widowed	26 (6.5)
Decline	7
Insurance Status, n (%)	
Private commercial	272 (67.5)
Medicare	114 (28.3)
Medicaid	12 (3.0)
Worker's Compensation	4 (1.0)
Motor vehicle	1 (0.2)
Decline	3
Smoking Status, n (%)	
Never	221 (54.6)
Former	127 (31.4)
Active	57 (14.1)
Decline	1

ADI, area deprivation index; BMI, body mass index.

### Univariable completion and participation results

The Table 2 shows univariable logistic regression models used to determine the associations between PROMs completion and participation and sociodemographic predictors. For models with predictors containing more than two levels, the overall type-III *P* value is provided along with the odds ratio *P* value. Black patients had significantly lower odds of completion compared to White patients (odds ratio [OR] = 0.55, *P* = .008). Additionally, Asian (OR = 0.13, *P* = .001) and Black (OR = 0.53, *P* = .013) patients had significantly lower odds of participation compared to White patients. Patients of a minority background (defined as any patient of Hispanic ethnicity or non-White race) had significantly lower odds of completion (OR = 0.57, *P* = .01) and participation (OR = 0.46, *P* = .001) compared to patients of nonminority backgrounds. Patients living in areas with higher deprivation (ie, ADI Groups 2 and 3) had significantly lower odds of completion (*P* = .002) and lower odds of participation (*P* = .012) compared to subjects living in areas with lower deprivation (i.e., ADI Group 1). ADI Group 2 had 56% lower odds of completion (*P* = .004) and 48% lower odds of participation (*P* = .044) compared to ADI Group 1, and ADI Group 3 had 49% lower odds of completion (*P* = .003) and 55% lower odds of participation (*P* = .004) compared to ADI Group 1. Lastly, for every increase in patient age by one year, the odds of completion decreased by 2% (*P* = .010), and the odds of participation decreased by 3% (*P* < .001).

**Table 2:** Patient-Reported Outcomes Measurement Information System (PROMIS) Completion and Participation

Demographic Variable	Completion OR^1^ (95% CI)	Completion OR, *P* Value	Type III *P* Value	Participation OR (95% CI)	Participation OR, *P* Value	Type III *P* Value
Sex			.349			.329
Female	-	-		-	-	
Male	0.82 (0.54–1.24)	.349		0.79 (0.50–1.26)	.329	
Age	0.98 (0.97–1.00)	∗.01	∗.01	0.97 (0.95–0.99)	∗<.001	∗<.001
BMI	1.01 (0.98–1.03)	.497	.497	1.00 (0.98–1.03)	.826	.826
ADI			∗.002			∗.012
Group 1	-	-		-	-	
Group 2	0.44 (0.25–0.77)	∗.004		0.52 (0.28–0.98)	.044	
Group 3	0.51 (0.32–0.80)	∗.003		0.45 (0.27–0.78)	∗.004	
Race			∗.029			∗.001
White	-	-		-	-	
Black	0.55 (0.35–0.85)	∗.008		0.53 (0.32–0.88)	∗.013	
Asian	0.27 (0.06–1.28)	.1		0.13 (0.04–0.45)	∗.001	
Other	0.00 (0.00–1)	.985		0.11 (0.01–1.25)	.076	
Ethnic Background			.711			.354
Non-Hispanic/Latino	-	-		-	-	
Hispanic/Latino	1.26 (0.38–4.19)	.711		0.55 (0.16–1.94)	.354	
Minority Status			∗.01			∗.001
Nonminority	-	-		-	-	
Minority	0.57 (0.38–0.87)	.01		0.46 (0.28–0.73)	∗.001	
Marital Status			.808			.915
Single	-	-		-	-	
Married	1.04 (0.65–1.67)	.877		1.13 (0.66–1.94)	.659	
Divorced	0.95 (0.46–1.96)	.894		1.01 (0.45–2.29)	.975	
Widowed	0.67 (0.27–1.69)	.398		1.42 (0.49–4.14)	.523	
Insurance Status			.938			.458
Private Commercial	-	-		-	-	
Medicare	0.86 (0.55–1.35)	.509		0.68 (0.41–1.12)	.128	
Medicaid	0.74 (0.22–2.51)	.624		0.83 (0.22–3.17)	.786	
Worker's Comp	1.47 (0.20–10.61)	.701		0.28 (0.04–2.01)	.204	
Motor vehicle	0.00 (0.00–1)	.987		0.8 (0.00–1)	.989	
Smoking Status			.673			.081
Never	-	-		-	-	
Former	1.15 (0.73–1.79)	.548		0.78 (0.47–1.31)	.346	
Active	0.86 (0.47–1.59)	.64		0.49 (0.26–0.92)	∗.026	

ADI, area deprivation index; BMI, body mass index.

(-) Reference value.

∗ Significant *P* value.

### Multivariable completion and participation results

**Figure 1:**
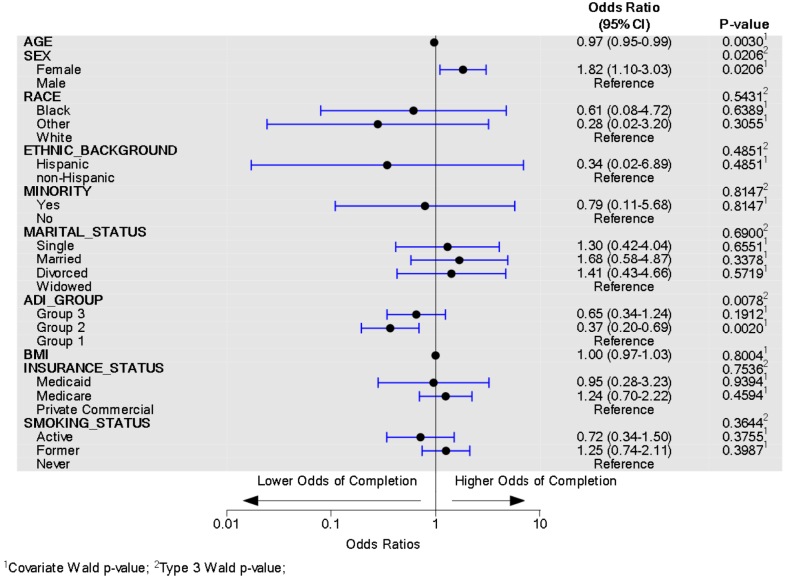
PROM completion multivariable logistic regression model forest plot.

**Figure 2:**
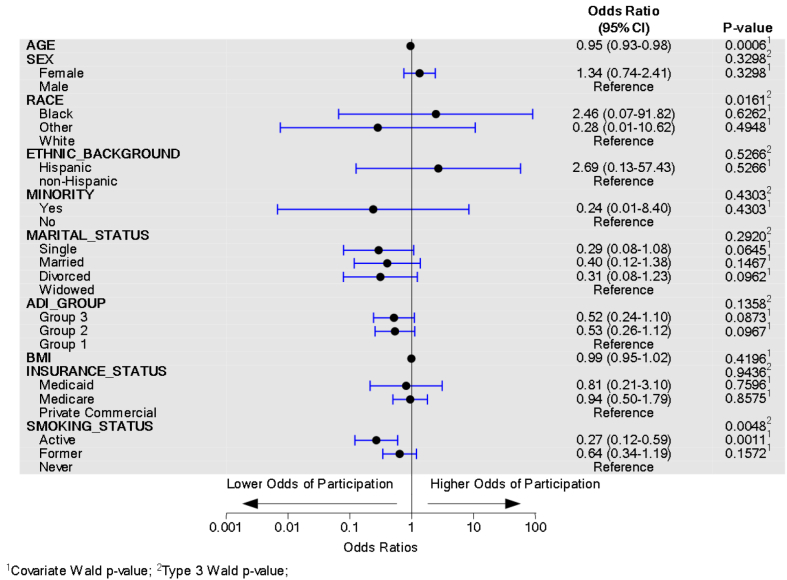
PROMs participation multivariable logistic regression model forest plot.

The Figures 1 and 2 display multivariable logistic regression model forest plots for PROMs completion and participation, respectively. To converge, some variable levels with small counts (race and insurance status) had categories combined. For race, the Asian and Other categories were combined into Other. Similarly, for insurance status, motor vehicle and worker’s compensation were combined into Medicaid. The model predicts the odds of completing all PROM surveys while controlling for all the included sociodemographic predictors. For variables with more than two levels, the overall type-III *P* value is provided along with the odds ratio and *P* value. PROMs completion (*P* = .003) and participation (*P* = .001) were significantly associated with age, such that for every increase in age by one year, the odds of completion decreased by 3% and odds of participation decreased by 5%, while controlling for other sociodemographic factors. Women were 1.82 times more likely to complete PROMs compared to men, while controlling for other sociodemographic factors (*P* = .021). Race was not associated with PROMs completion but was associated with PROMs participation (*P* = .016) while controlling for other sociodemographic factors; however, the individual comparisons with a reference category of White race were not significantly different. ADI group was significantly associated with PROMs completion (*P* = .008), with patients living in ADI Group 2 areas having significantly lower odds of PROMs completion compared to subjects living in ADI Group 1 areas, while controlling for other sociodemographic factors (OR = 0.37, *P* = .002). Smoking status and PROMs completion were not associated, but smoking status and PROMs participation were significantly associated (*P* = .005), with active smokers having significantly lower odds of participation compared to nonsmokers, while controlling for other sociodemographic factors (OR = 0.27, *P* = .001).

## Discussion

The purpose of this study was to determine the association between sociodemographic factors and PROMs survey completion and participation for hand surgery patients. Our hypotheses were supported in that PROMs completion and participation rates were significantly associated with sociodemographic factors, and sociodemographic factors associated with lower completion and participation rates are those that have been reported in orthopedic subspecialties. Patients with the following sociodemographic backgrounds were found to have lower completion and/or participation rates when controlling for other sociodemographic factors: older age (completion and participation), male sex (completion), Black race (participation), Asian race (participation), higher ADI groups (completion), and active smoking status (participation).

In comparing our results to those published in the literature, multiple studies similarly found Black race to be associated with lower PROMs completion rates.^2^^,^^4^^,^^6^ Older age was correlated with lower completion rates in a study of total joint arthroplasty patients by Schamber et al.^6^ Two studies—one for total joint arthroplasty patients and the other for spine surgery patients—found that residing in a lower income/higher distressed community was associated with lower completion rates.^2^^,^^7^ Active smoking status was also shown to be associated with lower completion rates in the same study of spine surgery patients.^2^

Although our study identified many of the same associations reported in prior studies, there were some discrepancies. Medicare/Medicaid insurance status was associated with lower completion rates in most previously published studies, whereas our study did not find insurance status to be associated.^4^^,^^6^^,^^7^ Nonmarried patients had a lower completion rate in the aggregate study from Bernstein et al,^4^ whereas marital status was not associated with completion rates in our study. Hispanic ethnicity was almost universally reported to be associated with lower completion rates previously but was not found to be associated in our study.^2^^,^^6^^,^^7^ Unlike our findings, the literature suggests that Asian race was not correlated to PROMs response rate, but this effect was mainly noted in joint arthroplasty patients.^7^^,^^12^

One possible reason for the differences between our data and those of the other mentioned studies could be because of the way in which completion or participation are determined. Schamber et al^6^ recorded participation as completion of at least one disease-specific and one generic survey up to 6 months before surgery and 5–18 months after surgery. Issa et al^2^ considered PROMs to be incomplete if the 1-year postoperative survey was not completed. Bernstein et al^4^ considered PROMs complete if all questions were answered for every assigned survey. Konopka et al^7^ considered PROMs complete for patients who filled out at least one survey at any before surgery or postoperative visit. For our study, we separated participation and completion, and used a combination of previously reported methods for determining participation or completion. As a result, some studies have more stringent criteria for measuring completion, whereas others have more lenient requirements, which produces variation in the data and analysis.

Multiple studies have speculated as to the causes of noncompletion or nonparticipation, citing technology barriers for older patients who may be less comfortable with technology, lack of access to technology for lower income patients, limited health literacy, and lack of in-office time to facilitate completion of surveys.^4^^,^^6^^,^^13^ One additional cause for incompletion that we noted in our study was loss to follow-up. Potential solutions to these barriers could include the medical assistant or front desk personnel asking patients if they need any assistance using the iPad and completing the survey and assisting as able; offering the surveys in additional languages for non-English speakers; care providers allowing extra time for patients to complete surveys before starting the patient interview; iPads being returned to a centralized location to be readily available and charged; and medical assistant or front desk personnel providing brief education about the purpose of the surveys. Sisodia et al^14^ suggested the most effective method for improving completion rates is through surgeon engagement and administrative support. Surgeon engagement would involve training physicians on the use of PROMs, and administrative support would include administrative surveillance of collection rates and the presence of a local clinical champion, who is a physician outside of the department who is the clinical contact for the Patient-Reported Outcomes program and oversees performance of the program. Other studies emphasized the importance of increased staff support to provide reminders, enroll patients in online portals, and monitor completion.^4^ There are also ongoing initiatives to translate PROMs surveys into languages such as Spanish, German, and Arabic, which would help mitigate language barriers.^15^ Issa et al^2^ recommended potentially creating a current procedural terminology code to bill for in-office PROM administration to compensate for the additional time required for in-office completion and incentivize surgeon investment in PROM collection. These solutions all address the barriers to completion for patients who attend follow-up appointments, but future studies should evaluate the role of loss to follow-up as it relates to PROMs incompletion, and consider possible solutions for this barrier.

Our study is subject to multiple limitations. The data for this study was collected from the hospital system EMR, and some patient profiles were incomplete or potentially included erroneous information. Additionally, there are no clear guidelines for selecting race and ethnicity within the EMR, and these variables may have been recorded inconsistently across patients. Another limitation comes from the implementation of PROM surveys at this health system in 2020. Although the surveys became available across the health system in 2020, it is unclear whether all patients who were included in this study were provided with the opportunity to complete PROM surveys at all visits in 2020. As such, some patient visits may have been recorded as a PROM completion opportunity when patients were not provided with a survey to complete. Another limitation of this study is the potential selection bias with the patient population if it did not reflect all sociodemographic backgrounds proportionately. Lastly, although this study identified a difference in completion and participation rates based on sociodemographic variables, we did not assess whether this discrepancy impacts functional outcomes or postoperative results. We also did not identify specific barriers to completion, and why these barriers exist. As such, future research would benefit from evaluating the barriers to PROM completion, and the potential impact of disparate completion/participation rates on patient outcomes.

This study found that PROM completion and participation rates were lower for hand surgery patients of older age (participation and completion), male sex (completion only), Black race (completion and participation), Asian race (participation only), racial/ethnic minority status (completion and participation), higher area deprivation group (completion and participation), and those with active smoking status (participation only). These data support similar findings within other subspecialties of orthopedics and support the need for equity with PROM collection to more accurately represent patients from all sociodemographic backgrounds.

## Conflicts of Interest

No benefits in any form have been received or will be received related directly to this article.
